# A Cross-Disciplinary Successful Aging Intervention and Evaluation: Comparison of Person-to-Person and Digital-Assisted Approaches

**DOI:** 10.3390/ijerph15050913

**Published:** 2018-05-04

**Authors:** Hui-Chuan Hsu, Tsuann Kuo, Ju-Ping Lin, Wei-Chung Hsu, Chia-Wen Yu, Yen-Cheng Chen, Wan-Zhen Xie, Wei-Chiang Hsu, Ya-Lan Hsu, Mu-Ting Yu

**Affiliations:** 1 Department of Health Care Administration, Asia University, Taichung 41354, Taiwan; shana3517@gmail.com (Y.-C.C.); bbb102031021@gmail.com (W.-Z.X.); johninnba@gmail.com (W.-C.H.); hsuya1993@gmail.com (Y.-L.H.); 2014yc@gmail.com (M.-T.Y.); 2 Department of Medical Sociology and Social Work, Chung Shang Medical University, Taichung 40201, Taiwan; tsuann@csmu.edu.tw; 3 Department of Human Development and Family Studies, National Taiwan Normal University, Taipei 10610, Taiwan; t10016@ntnu.edu.tw (J.-P.L.); queenfishqueen@gmail.com (C.-W.Y.); 4 Department of Radiation Oncology, Chung-Kang Branch, Cheng-Ching General Hospital, Taichung 40764, Taiwan; b751106@yahoo.com.tw

**Keywords:** cross-disciplinary, health promotion, intervention, successful aging

## Abstract

*Background*: Successful aging has been the paradigm of old-age life. The purpose of this study was to implement and evaluate a cross-disciplinary intervention program using two approaches for community-based older adults in Taichung, Taiwan. *Methods*: The content of the intervention included successful aging concepts and preparation, physical activity, chronic disease and health management, dietary and nutrition information, cognitive training, emotional awareness and coping skills, family relationship and resilience, legal concepts regarding financial protection, and Internet use. The traditional person-to-person (P2P) intervention approach was implemented among participants at urban centers, and the personal-and-digital (P&D) intervention approach was implemented among participants at rural centers; before the P&D group received the intervention, participants were assessed as the control group for comparison. *Results*: Healthy behavior and nutrition improved for the P2P group, although not significantly. Strategies for adapting to old age and reducing ineffective coping were significantly improved in the P2P group. The ability to search for health information improved in the P&D group, and knowledge of finance-related law increased in the P2P group. *Conclusion*: A continuous, well-designed and evidence-based intervention program is beneficial for improving the health of older adults, or at least delaying its decline.

## 1. Introduction

Successful aging has been widely accepted as the paradigm for the quality of life when growing older, and it is a multi-dimensional concept [1]. General risk and protective factors of successful aging have been found [2,3,4,5,6,7,8,9,10,11]. Effective intervention strategies to support successful aging have been accordingly developed. However, most health promotion intervention studies have focused on one aspect of successful aging, and only a few have considered a cross-disciplinary and multi-dimensional goal of intervention for successful aging [12,13,14]. In addition, Rowe and Kahn’s successful aging 2.0 idea [15] indicates that six aspects should be prioritized for policies focused on old age: intergenerational relationship, family (evolution, support, and role changes), productivity (work and retirement, function and dysfunction, technology, social role), human capital development (lifelong learning and skill training), and health and healthcare. These aspects indicate that a new approach for promoting successful aging should seek not only to increase the awareness of preparation for growing older and aging successfully, but also to increase the ability for lifelong learning, social participation, and health. Therefore, a comprehensive and prospective intervention should cover multiple dimensions of successful aging, including increased lifelong learning ability, and the model should be easily implemented across groups. In this study, we sought to conduct two different models of a successful aging intervention: one was the person-to-person (P2P) lecture model; the other was P2P plus the personal-and-digital (P&D) model. Both models were designed to be multi-dimensional and facilitate lifelong learning, but the P&D model also focused on an easily disseminated design. We sought to compare these two different intervention models to a control group and evaluate the effectiveness of the intervention in this study. 

### 1.1. Intervention on Successful Aging

There are many studies related to interventions for specific chronic diseases. For example, biomarkers or physical activity can be improved by exercise and diet community-based intervention [16,17,18,19,20,21], which works even for adults with developmental disabilities [22]. There are also some effective exercise interventions for specific physical function [23,24]. In addition, changes in lifestyle, health literacy, and healthy behaviors have been combined with physical health intervention programs, especially for the health management of chronic disease patients [16,17,18,25].

The goal of promoting mental health in older adults usually includes cognition, emotion, coping ability, and psychological well-being. Interventions for cognitive function use different strategies. For example, appropriate diet (nutrition) may reduce cognitive decline [26]. Cognitive training may improve cognitive function to delay its decline [27,28,29,30,31]. Computer-based training is as effective as paper-and-pen interventions for cognitive function, but computer-based training programs are less labor-intensive [32]. Physical activity interventions are also beneficial for cognition, memory, and visual/hearing abilities in older adults [33,34]. Regarding emotional health, using cognitive psychological therapy (such as acceptance and commitment therapy and mindfulness-based cognitive therapy) may strengthen mental resilience [35]. Life review [36], stress coping skills, and case management [37] may reduce depressive symptoms. Participation in an online support group may reduce depressive symptoms and increase dignity and quality of life [38]. Professional therapy and counseling using face-to-face education plus phone call reminders for self-care can also effectively increase mental health and self-care ability [39]. Face-to-face behavioral cognition therapy (relaxation and emotional regulation) as well as online videos will reduce symptoms of depression and anxiety. In addition, autobiographical memory training also increases life satisfaction and happiness [40]. 

Social activities, leisure activities, and social participation improve well-being [41]. Middle-aged people or adults preparing for retirement by anticipating social participation after this change find that maintaining their physical and mental states, as well as developing their social support network, are also useful [42,43]. In addition, family-centeredness is the core of Chinese culture. The family relationships affect the life satisfaction of older adults in Taiwan [44]. The health conditions of family members may affect not only older adults, but also family relationships [45]. However, most health promotion interventions for older adults related to family have focused on how to provide support for the patient’s family or how to reduce burden and stress for family caregivers [46,47,48], with scarce focus on increasing social activity engagement and positive support from family.

Financial security is often mentioned in retirement preparation [6,49], and financial security is one of the important components of successful aging for Taiwanese adults [50]. Financial management or preparation issues must consider legal issues related to heritage and economic security for those who are disabled or have dementia. However, these issues are hardly mentioned in intervention programs for older adults. 

### 1.2. Intervention Approach

As digital technology has developed, e-approaches and technology-assisted approaches have also been used in health education and intervention. Furthermore, the use of informational communication technology (ICT) has been viewed as an indicator of active aging [51] since using the Internet is a modern way for people to connect to their social network and participate in society. If an e-approach proves to be effective, such an approach will likely have the capacity for easy dissemination and replication in a wider range of the target population. Online courses or Internet social media groups are found to be effective for changing lifestyle or behaviors. For example, online courses can effectively change a diet and increase the intake of vegetables and fruits [52] or increase physical activities [53]. Online social networks may encourage social support and self-regulation of physical activity and diet [54]. 

The theoretical explanations supporting the application of social media for intervention include uses and gratification theory, common identity/common bond, social identity theory, social support theory, social network thresholds, diffusion of innovation, and so on [55]. A systematic review indicates that face-to-face interventions are still more effective than other interventional approaches, and their effectiveness depends on the characteristics of the target group [20]. Another systematic review found that a strengthened approach using a personal coach plus telephone or email is more effective [19]. The effectiveness of the intervention via online social groups depends on the characteristics of the members, including the composition, mutual support, and interaction [22,55]. If using a web-based self-management system after a face-to-face course without including a social group for strengthen, behavioral changes can be limited and may be resisted by some users [56]. The effectiveness of an eHealth intervention on mental health also depends on the types of intervention used, such as mindfulness training, cognitive behavior therapy, and stress management training [57].

The challenges for online intervention include how to measure the status and changes of the participants [58] and remaining aware that frequent participation online does not assure the desired change in healthy behaviors or health status [59]. When considering the development of an intervention program using ICT for older adults, the main barriers include the accessibility of broadband and websites, performance in learning, availability of training, motivation to use the Internet, and trust in technology [60]. Nevertheless, friendly displays may help to reduce the barriers of Internet usage [61], and smart phones or tablets that eliminate typing on keyboards are easier to use than computers. 

In Taiwan, health promotion programs for older adults are very common among communities. However, there is little intervention in the design of using ICT because older cohorts are less educated and use fewer ICTs than younger cohorts. Using ICT itself is the main barrier preventing access to lifelong learning materials on the Internet. In Taiwan, the percentage of older adults aged 55 or above using ICT to go online was approximately 60.4% in 2017 [13]. The use of ICT in middle-aged and younger cohorts is much more prevalent than that in older cohorts; the middle-aged and younger cohorts will be familiar with the Internet as they age in the forthcoming decade. Currently, to design and conduct a health promotion program available via ICT devices for current older adults is timely, but a friendly interface and person-assisted program is also important. 

### 1.3. Aims

In this study, we aimed to design and evaluate a successful aging promotion program for community-based older adults that is cross-disciplinary, enables lifelong learning, and is easily disseminated; to do this, we used two different models in Taiwan. Under the community care social welfare policy, there are community care centers (CCCs) run by not-for-profit organizations in almost all districts that are centrally located in neighborhoods. The CCCs provide different activities, health education, and scheduled courses for older adults in the community as a chance for social participation. In this study, we cooperated with multiple CCCs in Taichung, Taiwan to implement a successful aging intervention program and to evaluate its effectiveness. If the program proves to be useful, the model can be a reference for other CCCs to promote future health and successful aging. 

## 2. Materials and Methods 

### 2.1. Setting and Participants

**Figure 1:**
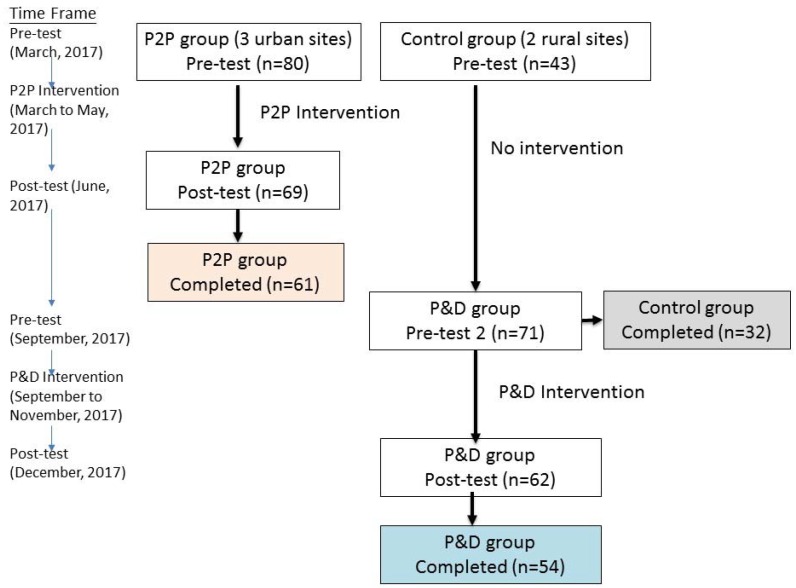
The study design and the participant flow. P2P: person-to-person; P&D: personal-and-digital.

The intervention was implemented in the CCCs, and all the designs had to conform to the daily routine for existing CCC participants and the CCC managers; thus, a randomized trial was not possible. The study was a quasi-experimental, multiple-center design for older adults in the communities. We contacted several rural and urban CCCs in Taichung. Three urban CCCs were run by a church-related organization, and two rural CCCs were run by two different social development associations; both agreed to participate in the program. The three church-led CCCs agreed to participate as the traditional person-to-person (P2P) experimental group in the face-to-face lecture-based intervention model, while the two rural CCCs agreed to participate in the program as the control group at the first stage and then as the person-and-digital (P&D) experimental group. All the existing older participants in the CCCs were invited to the program in 2017. Participants who were disabled, had dementia, or had difficulty with communication participated in the program but were excluded from the program evaluation. In addition, if there were newcomers to the CCCs during the intervention, they were excluded from the evaluation. Only those who participated in the intervention program and completed the pre- and post-test evaluations were included in the analysis. The participant flow and study design are described in Figure 1. There were 61 participants in the P2P group who completed the pre- and post-tests and the intervention in the three urban centers. The two rural centers were initially monitored as the control group without our intervention, but only their scheduled activities were accepted at the first stage. A total of 32 participants completed both the pre- and the post-tests at the first stage. The post-test at the first stage was used as the pre-test of the second stage, and the participants then received the P&D model intervention. There were 54 participants in the rural group who completed the pre- and post-tests in the P&D program.

### 2.2. Intervention Program

The activity times for the CCCs were every morning from Monday to Friday. Two of the urban center participants were grouped together for the intervention program. Thus, there were two intervention centers for the P2P intervention and two centers for the P&D intervention. The intervention program was given once per week, and the program lasted for at least 12 weeks for each center. The contents of the intervention for the two experimental groups were designed using the same construct, but the interventional approach was different. All the lecturers agreed to offer personal consultations after the intervention if the older adults had questions about health or aging issues. There were nine components included in the intervention design:

(1) Concept and preparation for successful aging: The lecture introduced the idea of successful aging, risk and protective factors to successful aging, strategies for successful aging, the importance of preparing for old age in life, and the framework of the whole program. The course was given by an expert in gerontology.
(2) Physical activity: The unit introduced content about common physical activities and a fitness examination and then provided a demonstration of easy physical activity exercises that can be performed at home. An elastic band was provided for each participant to use in the physical activity exercise. The unit was given by a physical therapist, and trained graduate students helped to demonstrate and assist the participants with the exercise.
(3) Nutrition and diet for older people: The educational content included reference material about daily diet, six categories of food and nutrition, portion size and calories, appropriate nutrition suggestions for older adults, the DASH diet (dietary approaches to stop hypertension, a diet which is rich in fruits, vegetables, whole grains, and low-fat dairy foods; includes meat, fish, poultry, nuts, and beans; and is limited in sugar-sweetened foods and beverages, red meat, and added fats; it is used for control hypertension and promote a well-balanced diet), etc. The lecture was given by an attending physician.
(4) Chronic disease prevention and management: This unit included the management of common chronic diseases for older adults (hypertension, high blood cholesterol, heart disease, diabetes, arthritis, osteoporosis, insomnia, etc.), prevention strategies, and medication usage knowledge. The lecture was given by an attending physician.
(5) Emotional health and coping with stress: The unit designed with the background of cognitive behavior therapy introduced self-awareness of emotions and distress, as well as practice of coping skills for a more balanced psychological well-being. The lecture was given by an expert and practitioner in the field of mental health.
(6) Cognitive function training: Cognitive function training was provided, including activities to draw attention and concentration, retain short-term memory, execute daily activities of daily living, and perform executive functioning tasks. A device-assisted response training was given for the P2P group, and an online cognitive game was implemented as the exercise for the P&D group. The necessary training devices and tablets were provided in the training weeks.
(7) Family relationship: The content focused on emotional awareness, forgiveness and conflict resolution in family relationships, and expressing appreciation and affection. The unit included a lecture and exercise, and a tablet game was also provided as the demonstration. The unit was led by two experts in family relationships.
(8) Financial security: The lecture focused on the basic ideas of self-protection concepts in finance, knowledge of law related to inheritance, legal representation for those who are disabled, and property trust. The lecture was given by a lawyer with expertise in social protection for older adults.
(9) Internet use: The lecture and practice focused on how to use a tablet to go online and introduced several useful applications for older adults, such as bus schedules, clinic registrations, and searching for information about songs on YouTube or health information on a recommended website. The tablets were provided for all the participants for the duration of the intervention. For the P&D group, the Internet use course was allowed additional weeks for practice. The lecture was given by a graduate student, and 4–10 students (according to the number of participants and the course content) and CCC volunteers assisted the older participants in learning to use the tablets every week.

For the same component of the intervention, the approaches used for the two experimental groups were different. The P2P group received traditional intervention education, that is, the educator (and graduate assistants) gave the lecture face-to-face every week at the two urban centers. The P&D group received face-to-face instruction combined with digital courses, and graduate students assisted in the learning activities. 

Each intervention component was given for at least one week, and the exercise was designed in the following weeks. For example, the physical activity exercise was conducted every week during the break; there were extra weeks for cognitive training exercise games and for using the tablets to go online. In addition, there were two different strengthening strategies for the two experimental groups. Participants in the P2P group were invited to join a Facebook group. All the intervention lectures or demonstrations were recorded as videos, uploaded online, and then embedded in the Facebook group interface. The participants were invited to watch the videos after the program so that they could review the videos or catch up in case they missed the lecture. For the P&D group, participants were asked to complete reminder questions online before class every week. The questions were related to the frequencies of engaging in physical activity, eating vegetables and fruit, expressing appreciation, showing gratitude to others, engaging in cognition related activities, and other topics in the past week. All the items focused on the intervention content. 

### 2.3. Ethical Consideration

All participants were introduced to the intervention program, the contents of the evaluation questionnaire, and the fitness items. They were invited to participate in the program and complete the questionnaire evaluation, and they could decline to participate in the research project. All the researchers and students involved in this study were required to sign agreements for the protection of the privacy of the participants. Assurances were given that all data would be presented as anonymous statistics and that privacy would be protected. The study was conducted in accordance with the Declaration of Helsinki, and the protocol was approved by the Medical Research Ethics Committee of Asia University (No. 10411015). 

### 2.4. Data Collection and Measurements of Effective Evaluation

Two experts with a background in gerontology and health promotion reviewed the questionnaire for content validity, and the questionnaire was pretested and accordingly revised before it was administered to the study participants. The variables for evaluating the effects of the intervention program included:

(1) Demographics and background: Age, sex, education, marital status, living arrangement (living alone or with others), household income, and rated relative income.
(2) Physical health: Self-rated health was measured as poor to excellent (score 1–5). Chronic disease numbers (accumulative numbers of the following chronic diseases: strokes (ever), hypertension, heart disease, diabetes, arthritis, spinal spurs, kidney or urinary tract diseases, stomach ulcers, liver- or gall bladder-related diseases, pulmonary or respiratory system diseases, cancer, cataracts or glaucoma, and reproductive system diseases). Nutritional risk was screened by the Mini Nutrition Assessment (MNA) [62] and coded as normal (score ≥ 12) or at risk of malnutrition (score ≤ 11). Physical function, measured by instrumental activities of daily living (IADLs) and the Nagi scale and scored 0–27 [63], was collected only at baseline to serve as a control. Fitness items for older people were also examined as an outcome but were not included in this article because some participants could not perform the fitness tests, and the sample size was small.
(3) Mental health: Depressive symptoms were measured by the CES-D 10-item scale [64], scored 0–30. Cognitive function was measured by MoCA (Montreal Cognitive Assessment) [65], a more sensitive cognitive assessment tool; the total score ranged from 0 to 30 by the adjustment of education. A score of 25 or lower indicated the risk of mild cognitive impairment. Adaptation strategies to old age were conceptualized from the Baltes and Baltes’ successful aging model [66] and revised by Donnellan et al. (2012) [67]. The six items included the strategies of selection, optimization, and compensation. Each item was scored from 1 to 3 as disagree, no opinion, or agree; each type of strategy was scored from 2–6. Stress coping strategies were measured with a brief COPE scale [68]. The original scale included 18 items and was then reduced to 11 items in later tests. The stress coping strategies were analyzed by factor analysis, and three types of coping strategies were then extracted. Finally, the original item scores were summed as the strategy type score: action and positive thinking (score 4–16), emotion-focused coping (score 4–16), and negative coping (score 2–8).
(4) Lifestyle and health literacy: Lifestyle included regular exercise (exercise three times or more per week), sedentary lifestyle hours per day, etc. In addition, 14 items for measuring diet behaviors and healthy literacy were asked [13], such as questions about choices for a balanced diet; intake of vegetables, fruit, milk and related products, drinks containing sugar, and foods high in cholesterol; noticing expiration dates; reminders from family and friends about diet nutrition, etc. Each item was scored from 1 to 3, and the total score ranged from 14 to 42; a higher score indicated a better diet style.
(5) Social support: (a) Social support included questions about emotional support and instrumental support from family and friends, respectively; each item was scored from 4 to 12; (b) conflicts with family members (including spouse, children, or other family members) (coded as yes/no).
(6) Financial security knowledge and preparation: The participants were asked if they had made financial preparations for life in old age (yes/no). They were also asked whether they knew about the laws regarding inheritance, guardian, and property trust (yes/no).
(7) Internet use: The participants were asked whether they use the Internet and at what frequency (at least once every week) as well as whether they were able to search for health information online (yes/no). In the post-test, participants were also queried about reasons to use or not use the internet.
(8) Life satisfaction: Life satisfaction was scored from 1 to 5, indicating very unsatisfactory to very satisfactory

### 2.5. Analysis

First, we compared the differences across groups at baseline in Table 1 by descriptive analysis, chi-square test, and one-way ANOVA. If there were group differences at baseline and natural time changes, group differences needed to be controlled. Next, the changes within groups were compared by McNemar test and paired-t test in Table 2, that indicated the changes over time of the same older participants. Health status and health behaviors may change over time even though no intervention was conducted, especially for the frail older people. Thus, generalized estimating equation (GEE) [69] was used for controlling the baseline group differences and natural time changes within group to examine the real effects of interventions. 

In addition, a process evaluation was needed to provide qualitative descriptions of how the program was conducted and to explain the outcome (please see the Discussion section). According to the framework of Steckler and Linnan [70], the process evaluation included the following components: recruitment, maintenance, context, resource, implementation, reach, barriers, exposure, continued use, and contamination. 

## 3. Results

### 3.1. Sample Description

Table 1 shows the characteristics at baseline across the three groups. The education level of the urban area (P2P group) was higher than that of the rural area groups, and ethnicity and religious beliefs were significantly different between the urban and rural areas. 

### 3.2. Comparison within Groups

Table 2 shows the changes of the outcome variables before and after intervention within the two experimental groups and the control group. For the P2P group, the self-rated health improved, nutrition risk improved, negative coping strategies reduced, and financial security knowledge increased significantly. For the P&D group, diet behavior and diet literacy increased, use of selection adaptation strategy for aging increased, and life satisfaction increased significantly. Other changes were observed but were not significant. Some changes also were observed in the control group: their self-rated health increased, diet behavior improved, selection adaptation strategy to aging increased, and financial preparation percentage increased. It seems there were group differences at baseline that may interfere with the evaluation in a quasi-experimental design. Thus, generalized estimating equation models were necessary to control the group differences. Table 3 show the results of generalized estimation equation models to examine the intervention effects by controlling the groups, time, age, sex, and education. The group effect indicated the difference between the experimental group (P2P or P&D) and the control group; the time effect indicated the change over time before and after intervention. The group and time interaction term indicated the real intervention effect for the P2P group or for the P&D group.

### 3.3. GEE Analysis

Table 3 shows the results of GEE models used to examine the intervention effects by controlling for the groups, time, age, sex, and education. The group effect indicated the difference between the experimental group (P2P or P&D) and the control group; the time effect indicated the change over time before and after the intervention. The group and time interaction term indicated the real intervention effect for the P2P group or the P&D group. 

The first part of Table 3 shows the intervention effects on physical health and health behaviors. Only nutrition risk was significantly reduced for the P&D group compared with the control group (β = −0.073, *p* < 0.05); the P2P group was not significant. The change in self-rated health was small. Regular exercise increased, and sedentary activity decreased after the intervention in the two experimental groups, but these changes were not significant. Diet behavior and diet literacy increased more for the P&D group than for the P2P group compared with the control group, but not significantly. 

The second part of Table 3 shows the intervention effects on mental health, adaptation strategies for aging, and strategies for coping with stress. Cognitive function increased for both the P2P and P&D groups, although this was not significant. Cognitive function increased over time (β = 0.112, *p* < 0.01) for all groups, showing that the learning effect may exist. Depressive symptoms were also not significant. Among the three adaptation strategies for aging, only the selection adaptation strategy significantly increased for both the P2P and P&D groups. Regarding the three types of strategies for coping with stress, the only significant effect observed was for reducing the use of emotion-focused coping for the P2P group. The P&D group increased its use of the action-and-positive-thinking strategy and the emotion-focused strategy, but not significantly. 

The third part of Table 3 shows the intervention effects on social and economic dimensions and life satisfaction. Expectedly, the family support rating was reduced for the P2P group. The support from friends was significantly changed. Conflicts with family members for both the P2P and P&D groups were reduced, although this was not significant. The use of the Internet increased for both experimental groups, although the coefficients were not significant. However, the P&D group significantly increased its ability to search for health information online. Knowledge about financial security law increased in the P2P group. Life satisfaction was not significant. 

## 4. Discussion

### 4.1. Summary

This study used two different interventional approaches—face-to-face P2P lectures and a person-plus-digital course—to conduct a cross-disciplinary study on successful aging promotion programs for community-based older adults in a community care center setting in Taiwan. The significant changes brought about by the intervention included reduced risk of unhealthy nutrition, increased selection adaptation, reduced reliance on emotion-focused coping, reduced family support, an increased ability to search for health information, and increased financial security and knowledge. Nutritional status, adaptation to aging, and searching for health information online especially worked better for the P&D group than for the P2P group. A digitally assisted and student-led intervention approach can be effective in a way that can be replicated and widely implemented.

### 4.2. Outcome Evaluation

#### 4.2.1. Physical Health and Health Behaviors

Unlike in previous studies, the physical health outcome (self-rated health, fitness, and physical function) was not significantly improved in this study. Because the participants were older, their physical functions could likely not be improved as much in such a short time compared with those of middle-aged people [13]. Among the healthy behaviors, regular exercise increased and sedentary activity hours decreased but were insignificant, as in previous studies [17,18,25,29,53], and the habits of smoking or drinking also did not significantly change. It is possible that the existing CCCs often provide physical activity programs; the P2P and P&D groups already had the chance to do sufficient physical activities before the intervention program. Knowledge and behavior of a healthy diet showed a larger increase for the P&D group than for the P2P group, and the risk for poor nutrition for the P&D group was significantly reduced. The P2P group received similar nutrition knowledge in courses prior to the intervention, and their diet behavior and knowledge were possibly not very altered after the intervention. The effects of increased dietary knowledge and improved behavior, as well as reduced nutrition risk, may have come from our intervention program, especially from the easier to grasp but important core knowledge we designed. There was another nutrition-related intervention program conducted in the same rural area, and it is possible that participation in that program also affected the knowledge of the P&D group; all the improvement may not have been due to our effort. Nevertheless, the results show that dietary behavior and nutrition statuses can be changed by health education. 

#### 4.2.2. Mental Health

This intervention program provided cognitive training for both the experimental groups. Although the change was not significant, the cognitive function for both groups increased; the change for the P&D group (β = 0.132) compared with the control group, especially, was larger than that of the P2P group (β = 0.055). The P&D group participants had less education and lower cognitive function at baseline than the P2P group (β = 2.840, *p* < 0.05). The content of cognitive training was similar for the second experimental group, and the intervention approach was both by face-to-face lecture and practice. The only difference was that the P&D group had extra practice with the cognitive-training game online, using the tablets we provided after the lecture. Cognitive training may work for less educated people, or the extra practice of cognitive training has a strengthening effect on cognitive function. 

Depressive symptoms were not significantly altered by the intervention on coping skills, as shown in other research [37]. However, mental well-being and positive emotions significantly increased for the P&D group. This part is not included in the current paper, since only the P&D group was measured. Selection strategies for adaptation to aging increased for both the P2P and P&D groups after the very first part of intervention, which presented information about successful aging concepts and strategies. Selection refers to an individual focusing attention on fewer, more important goals, and is the first step that older adults should take to adapt to aging by either elective or loss-based choice [66]. After the course on emotion awareness and stress management, there was a significant reduction in coping strategies using an emotion-focused strategy for the P2P group; this did not occur with the other coping strategies. It is possible that the experimental groups gained awareness of their own emotions and deeper psychological needs and thus experienced less emotion fluctuation and more stability than before; emotion-focused coping was less needed. 

#### 4.2.3. Social Outcome

An unexpected finding was that self-perceived family support for the P2P group diminished after the intervention. There were some participants in the P2P group who did not have a spouse or children, and family may play only a smaller part in their lives. It is possible that social networks and support from the community, such as the staff and friends in CCCs, may be even more important and became their second family; it is also possible that some important confounders were not controlled for in the model, such as family composition. The reason for the reduced perceived family support needs to be explored in further study. 

The knowledge-based intervention showed effectiveness, especially in learning about Internet use and financial security. Both experimental groups were given a course and practice using the Internet; the P&D group, in particular, was given an Internet class for a month and practice that continued for 3 months. Both groups increased their habit of Internet use (at least once a week), but not significantly. However, the ability to search for health information online was significantly increased for the P&D group, despite their lower educational level compared with P2P group. Although the P&D group participants did not have experience in using the Internet or cell phones, their ability to use these devices for health information was improved with the assistance of students and continued practice. The intervention is expected to be useful in promoting eHealth information or health literacy in the future. The lecture about finance protection and economic security also proved to work in the face-to-face lecture format for the P2P group. A video-assisted and student-led discussion about finance-related law may not be enough, and an expert-led discussion to disseminate basic concepts of financial security may be more useful. 

### 4.3. Process Evaluation and Intervention Approach

The P2P group was conducted in the urban CCCs under the supervision of a church charity organization. The strengths of these urban CCCs were that the facility was sufficient and there were enough volunteers to help with the program. One of the invited urban CCCs promised to participate but did not do so at all; thus, the number of participants was less than expected. The education level of the urban CCCs was higher than that of the rural CCCs, and the courses for the urban CCCs before this intervention program were given systematically by the organization. The baseline scores of the P2P group were better in education and health literacy than those for the rural areas (the P&D group and the control group). The lifelong learning atmosphere was better in the P2P group, and those participants demanded advanced knowledge and skills in health promotion. Thus, the dose and reach of the intervention for the P2P group was more advanced than what was provided for the rural P&D group. It was unfortunate that the church charity organization did not want to participate in the digital intervention and preferred the traditional face-to-face intervention. Thus, the Internet and tablet practice course for the P2P group was shorter. 

In the original study design, the P2P group was to be assessed with two post-tests to gauge the continued use and shorter effect (first post-test) and longer effect (second post-test) at 3 months after intervention. However, the Taiwanese government announced it was implementing a related physical and cognitive disability program and encouraged CCCs to participate. Two urban CCCs wanted to participate in the program after the first post-test. If we continued to collect data for the second post-test, the effect could not be differentiated from the other intervention program. Thus, we decided to give up the second post-test data collection for the P2P group. 

The P&D group and the control group were from the same rural area. The educational level of the participants in the rural area was lower. In addition, many older participants in the rural area did not use a cell phone, let alone a smart phone. Thus, the content for the digital course needed to be shorter and more basic. Therefore, the dose of the intervention was lower for the P&D group than for the P2P group. There were barriers to implementing the P&D intervention in the rural CCCs. For example, some of the older adults in the P&D group were illiterate, and they may have resisted learning to use the ICT at the beginning. It took a longer time for these subjects to become familiar with the interface without typing Chinese characters. Fortunately, the volunteers helped to teach the use of tablets, and the practice of playing games or watching videos on YouTube successfully captured the interest of the participants. In addition, the rural older adults were friendly and very welcoming toward the lecturers and students who were implementing this program. The companionship of the students and the new knowledge and skills acquired from the program made the older participants feel current, interested, and satisfied. However, the reach of the ICT learning use was less than we expected. We provided tablets for the experimental group participants to practice. However, since the tablets were limited, the participants were not engaged. Some individuals tried to get tablets but were unwilling to participate in the intervention. Thus, the tablets we offered were stored in the CCCs and could be used only while the participants were at the sites; thus, participants were unable to take the tablets home for practice. In addition, we found that the participants in the rural CCCs showed a less-structured style of participation. They did not necessarily come or leave the site on time, and they concentrated less while in the lecture and were more interested in physical activities. The intervention effect may have been lower than intended by the original design. 

There were also unexpected interferences in the P&D and control groups. When we contacted the CCCs in the rural area, although there were other courses and plans being conducted in these CCCs at the same time, the content was not related to health or successful aging promotion. However, while the study started the intervention in the P&D group, these CCCs concurrently received another intervention program related to health education, nutrition, and healthy diet. The intervention effect for the P&D group was likely contaminated. The CCCs needed to arrange everyday courses for older participants, and our study could not afford to provide such intensive courses due to limited budgets and human resources. In addition, CCCs in Taiwan have become the most popular spots for researchers or the government to demonstrate topics for older adults. Contamination was unavoidable.

### 4.4. Strengths and Limitations

There are some strengths of this study. First, multiple outcome-based and process evaluations were conducted through the intervention. Many health promotion programs in Taiwan were conducted in the various CCCs; however, few applied appropriate evaluations of their effectiveness, or the design of the activities were not theory-based. There was not enough evidence for the effectiveness of the program given by CCCs to the older adults. The multiple dimensions of health, knowledge, behaviors, and attitudes were evaluated in this study. Such evidence-based intervention is needed and suggested for implementing health promotion programs in the community. Second, the intervention program for successful aging was cross-disciplinary. Most health promotion programs for older adults are focused on physical health, such as physical activity or disease management education. However, successful aging is multi-dimensional and, accordingly, multi-dimensional intervention should be designed and implemented. Our research team also included investigators from different bio-psycho-social backgrounds. Through dialogue among different disciplines and a well thought-out program sequencing, a multi-dimensional intervention program can be designed. Third, we developed two different intervention approaches for successful aging: the face-to-face lecture (P2P) and the person-plus-digital course (P&D). The experiment on using the Internet as assistance in intervention worked for very few older adults, since many did not have much experience using the Internet. The use of ICT itself is an indicator of active aging, and learning to use the Internet helps older adults to search for health information, provides practice on cognitive training, and offers the chance to connect with their family and friends. The results of the two different approaches offer evidence in intervention comparison and suggestions for future research or practice in the community. Fourth, we developed the education materials, and all the lectures were video-recorded. The successful aging material can be provided through the Internet. Fifth, the participants who were at risk for poor nutrition were screened, detected, and offered names of CCC managers for further care. Sixth, older adults with high risks of depression and cognitive decline were screened and detected. Referrals for further evaluations or family consent helped older adults stay active while getting care treatments. Seventh, many students participated in this program as teaching assistants for older adults. The students gained experience in conducting and evaluating an intervention program on successful aging, interviewing, and building relationship with older adults, as well as the knowledge of successful aging transformations for older people in the community. 

There are also some limitations in this study. First, a randomized clinical trial design was impossible in the community setting. We needed to respect the willingness of the CCCs regarding the intervention practice (time, frequency, methods, etc.). Therefore, there were differences among these CCCs regarding the P2P, P&D, and control groups. However, we controlled for the group differences in the GEE models. Second, the human resources required were demanding with such a multiple center design. Both experimental group interventions needed a lot of human (student) resources for leading and assisting the lectures and practices, especially for using the ICT. We managed to maintain the same team of instructors and experts for both P2P and P&D for quality purposes. However, the students in this program also had their own classes in the university during the semester, and the CCC schedules affected their spare time. Third, there were other programs continuing at the CCCs at the same time. Contamination may be an issue, and we may not be able to differentiate where the effects come from. Fourth, there was an unexpected policy in encouraging all the CCCs to conduct physical and cognitive disability interventions that were similar to ours. Therefore, we were unable to monitor the longer effect of the intervention to avoid duplication.

## 5. Conclusions

This cross-disciplinary intervention program for successful aging showed effects on physical and mental health, healthy behaviors, knowledge of the Internet, and financial security. Lifelong learning is helpful to promote health and successful aging for older adults. However, it may take time for the older adults to develop the group dynamics to appreciate such multi-disciplinary activities. Thus, health promotion should be offered early in life because the effect is even more significant, especially on physical health. Interventions on delaying disabilities or cognitive impairment could be effective for older adults, but these should be provided as early as possible to gain a better outcome. In addition, both the face-to-face and digital intervention approaches can be effective, but digital courses for older people require assistance and pretraining in the use of ICT devices, and some kinds of intervention still show better effects with face-to-face interaction. A model that is easily implemented across groups to design successful aging intervention programs needs to educate and cultivate seed leaders in the community for wider dissemination. In addition, an evidence-based and theory-based intervention program design is necessary to promote such interventions in all communities. 

## Figures and Tables

**Table 1:** Demographics of the participants at baseline (mean (SD) or %).

Variables	P2P (n = 61)	P&D (n = 54)	Control (n = 32)	Significance
Age	77.25 (8.27)	76.57 (7.01)	78.16 (7.07)	
Sex				
Male	16.7%	25.9%	28.1%	
Female	83.3%	74.1%	71.9%	
Education				
Illiterate	10.0%	20.4%	18.8%	*
Elementary school	30.0%	42.6%	40.6%	
Primary high school	23.3%	18.5%	28.1%	
Senior high school	21.7%	16.7%	12.5%	
College/University +	15.0%	0.0%	0.0%	
Marital status				
No spouse	63.3%	59.3%	53.1%	
Having spouse	36.7%	40.7%	46.9%	
Ethnic groups				
Mingnan	63.3%	88.9%	90.3%	**
Hakka	5.0%	1.9%	6.5%	
Mainland provinces	30.0%	9.3%	3.2%	
Aboriginal and others	1.7%	0.0%	0.0%	
Religion belief				
None	15.0%	7.4%	6.3%	***
Buddhism, Dao, folk belief	41.7%	85.2%	87.5%	
Christian, Catholics	43.3%	7.4%	6.3%	
Don’t know/Refused to answer	19.0%	46.9%	33.3%	
Relative income to others				
Higher	5.2%	0.0%	5.6%	
About the same	69.0%	61.3%	75.9%	
Lower	25.9%	38.7%	18.5%	
Living arrangement				
Alone	18.3%	25.0%	20.4%	
With others	81.7%	75.0%	79.6%	
Family not living together				
No	17.2%	6.3%	9.3%	
Yes	82.8%	93.8%	90.7%	

Note: chi-square test or one-way ANOVA test were conducted. * *p* < 0.05, ** *p* < 0.01, *** *p* < 0.001.

**Table 2:** Evaluation of the outcome variables before and after intervention within three groups.

Evaluation	P2P Group		P&D Group		Control Group	
Variables	Pre-test	Post-test	Pre-test	Post-test	Pre-test	Post-test
Self-rated health	3.10 (0.75)	3.38 (0.78) *	3.31 (1.01)	3.33 (0.91)	2.84 (0.99)	3.31 (1.18) *
Regular exercise (%)	77.90%	88.10%	72.20%	77.80%	76.60%	80.00%
Sedentary activity hours	4.69 (3.08)	5.03 (3.76)	5.58 (4.58)	4.25 (2.40)	4.97 (2.50)	5.73 (4.30)
Diet behavior and diet literacy	30.87 (4.76)	31.75 (4.34)	31.95 (4.47)	33.42 (5.16) *	30.52 (3.75)	32.32 (4.29) *
No nutrition risk (%)	82.5%	96.5% **	88.9%	87.0%	90.0%	86.7%
Cognitive function	22.91 (5.01)	23.37 (5.51)	17.94 (6.09)	18.86 (6.08)	17.89 (6.58)	18.39 (5.94)
Depressive symptoms	5.92 (5.06)	5.53 (4.67)	4.87 (5.05)	5.04 (5.26)	5.17 (5.91)	5.13 (6.46)
Adaptation: selection	5.05 (1.13)	5.13 (1.13)	4.25 (1.95)	4.90 (1.09) **	4.87 (1.31)	4.13 (1.07) **
Adaptation: optimization	5.65 (0.66)	5.42 (0.87)	5.33 (0.86)	5.44 (0.98)	5.63 (0.62)	5.40 (0.81)
Adaptation: compensation	5.22 (1.15)	4.98 (1.24)	5.21 (1.29)	5.27 (1.17)	5.23 (1.17)	5.37 (1.16)
Action and positive thinking coping	3.18 (0.64)	3.10 (0.78)	2.73 (0.90)	3.00 (0.73)	2.51 (0.96)	2.88 (0.93)
Negative coping	2.74 (0.60)	2.37 (0.72) **	2.25 (0.71)	2.51 (0.72)	2.34 (0.75)	2.28 (0.71)
Emotion-focused coping	1.90 (0.69)	1.77 (0.75)	1.63 (0.70)	1.66 (0.76)	1.62 (0.80)	1.53 (0.65)
Family social support	9.92 (1.99)	9.68 (1.96)	9.85 (1.88)	10.13 (1.93)	9.15 (2.34)	9.92 (1.76)
Friend social support	7.69 (1.44)	7.45 (1.50)	8.57 (1.63)	8.80 (1.60)	7.68 (1.82)	8.32 (1.49)
Conflict with family members	14.3%	4.8%	13.0%	4.3%	8.3%	8.3%
Use of Internet	23.7%	30.5%	15.7%	17.6%	10.3%	6.9%
Search for health information	20.3%	22.0%	40.0%	66.7%	6.9%	6.9%
Financial preparation	61.0%	62.7%	58.8%	58.8%	31.0%	62.1% *
Financial security knowledge	27.1%	45.8% *	25.5%	29.4%	24.1%	20.7%
Life satisfaction	3.88 (0.67)	4.02 (0.66)	3.90 (1.05)	4.31 (0.84) **	3.34 (0.23)	3.72 (0.22)

Note: McNemar test and paired-*t* test were conducted within the groups. * *p* < 0.05, ** *p* < 0.01.

**Table 3:** Effects on physical, mental, and social outcomes by the intervention program by generalized estimating equation (GEE) analysis.

**Physical Health and Health Behaviors**	**Self-Rated Health**	**Regular Exercise**	**Sedentary Activity Hours per Day**	**Diet Behaviors and Diet Literacy**	**No Nutrition Risk**			
Intercept	3.362 (0.694) ***	2.839 (2.061)	7.119 (2.578) **	26.841 (3.644) ***	4.983 (2.369) *			
P2P Group	0.165 (0.189)	0.121 (0.549)	−0.254 (0.653)	−0.727 (0.904)	−0.552 (0.706)			
P&D Group	0.448 (0.214) *	−0.214 (0.528)	0.341 (0.782)	1.335 (0.870)	−0.116 (0.728)			
Time (months)	0.081 (0.032) *	0.016 (0.086)	0.092 (0.141)	0.294 (0.125) *	0.014 (0.125)			
P2P * Time	0.008 (0.048)	0.189 (0.153)	−0.010 (0.237)	1.490 × 10^−16^ (0.235)	0.573 (0.251)			
P&D * Time	−0.074 (0.049)	0.084 (0.131)	−0.502 (0.264)	0.219 (0.248)	−0.073 (0.200) *			
Age	−0.006 (0.008)	−0.021 (0.025)	−0.022 9).029)	0.004 (0.042)	−0.033 (0.029)			
Sex (Female)	−0.390 (0.184) *	−0.112 (0.428)	0.534 (0.544)	2.448 (0.863) **	−0.348 (0.609)			
Education	0.163 (0.054) **	−0.004 (0.172)	−0.420 (0.209) *	1.196 (0.297) ***	−0.002 (0.207)			
QIC #	236.676	315.408	3409.545	5281.812	207.184			
**Mental Health**	**Cognitive Function**	**Depressive Symptoms**	**Adaptation: Selection**	**Adaptation: Optimization**	**Adaptation: Compensation**	**Coping: Action and Positive Thinking**	**Coping: Negative**	**Coping: Emotion-Focused**
Intercept	29.936 (5.246) ***	3.371 (4.107)	4.341 (0.767) ***	5.232 (0.595) ***	6.984 (0.922) ***	2.863 (0.577) ***	2.699 (0.477) ***	2.192 (0.489) ***
P2P Group	2.840 (1.331) *	0.958 (1.240)	0.165 (0.280)	−0.058 (0.131)	0.013 (0.255)	0.543 (0.188) **	0.227 (0.168)	0.406 (0.156) **
P&D Group	−0.049 (1.268)	−0.385 (1.243)	−0.554 (0.294)	−0.327 (0.156) *	−0.059 (0.271)	0.169 (0.204)	−0.082 (0.173)	−0.076 (0.162)
Time (months)	0.112 (0.132) **	−0.028 (0.191)	−0.122 (0.042) **	−0.039 (0.025)	0.006 (0.046)	0.064 (0.037)	−0.013 (0.034)	−0.008 (0.029)
P2P * Time	0.055 (0.227)	−0.100 (0.285)	0.150 (0.076) *	−0.039 (0.048)	−0.083 (0.075)	−0.085 (0.053)	−0.029 (0.049)	−0.114 (0.044) **
P&D * Time	0.132 (0.231)	0.154 (0.247)	0.323 (0.078) ***	0.061 (0.058)	0.004 (0.076)	0.023 (0.059)	0.018 (0.058)	0.081 (0.054)
Age	−0.205 (0.060) **	0.032 (0.049)	0.002 (0.008)	0.001 (0.007)	−0.021 (0.010) *	−0.009 (0.007)	−0.013 (0.006) *	0.000 (0.056)
Sex (Female)	0.677 (1.049)	0.131 (1.038)	0.234 (0.216)	0.458 (0.145) **	0.014 (0.207)	0.402 (0.140) **	0.122 (0.090)	0.230 (0.108) *
Education	2.507 (0.376) ***	−0.471 (0.379)	0.089 (0.067)	0.023 (0.043)	−0.079 (0.076)	0.079 (0.046)	−0.051 (0.038)	−0.019 (0.041)
QIC #	6540.177	7782.505	398.338	199.566	423.882	186.490	158.808	151.390
**Social Outcome**	**Family Social Support**	**Friend Social Support**	**Conflict with Any Family Members ^#^**	**Use of Internet**	**Search for Health Information**	**Financial Preparation**	**Financial Security Law Knowledge**	**Life Satisfaction**
Intercept	8.963 (1.588) ***	7.829 (1.295) ***	5.900 (4.806)	7.556 (2.456) **	7.157 (2.583) **	1.781 (1.617)	1.423 (1.726)	4.213 (0.671) ***
P2P Group	0.492 (0.459)	−0.075 (0.398)	0.806 (0.976)	0.536 (0.791)	0.852 (0.833)	0.935 (0.512)	−0.302 (0.516)	0.441 (0.218) *
P&D Group	0.514 (0.457)	0.796 (0.395) *	0.119 (0.980)	0.383 (0.825)	0.776 (0.867)	1.038 (0.527) *	−0.007 (0.518)	0.464 (0.247)
Time (months)	0.127 (0.063) *	0.065 (0.055)	−0.080 (0.100)	−0.101 (0.163)	−0.004 (0163)	0.180 (0.083) *	−0.045 (0.073)	0.048 (0.036)
P2P * Time	−0.206 (0.096) *	−0.159 (0.099)	−0.531 (0.356)	0.278 (0.199)	0.068 (0.198)	−0.139 (0.132)	0.344 (0.142) *	−0.003 (0.047)
P&D * Time	−0.050 (0.123)	−0.006 (0.099)	−0.213 (0.368)	0.160 (0.208)	0.884 (0.305) **	−0.222 (0.159)	0.108 (0.158)	0.073 (0.061)
Age	0.003 (0.019)	0.003 (0.015)	−0.094 (0.058)	−0.152 (0.031) ***	−0.159 (0.031) ***	−0.034 (0.019)	−0.049 (0.021) *	−0.008 (0.008)
Sex (Female)	−0.137 (0.301)	−0.610 (0.261) *	−0.622 (0.733)	0.149 (0.543)	−0.168 (0.657)	−0.193 (0.360)	0.221 (0.421)	−0.315 (0.171)
Education	0.166 (0.140)	0.122 (0.099)	−0.336 (0.417)	0.873 (0.210) ***	1.105 (0.257) ***	0.226 (0.125)	0.678 (0.172) ***	0.050 (0.056)
QIC #	1036.473	637.722	103.719	207.208	159.629	388.300	316.777	239.243

Note 1: The reference groups of the categorical variables were intervention (control group), sex (male), regular exercise (no), nutrition risk (no), any family conflict (no), use of Internet (no), search for health information (cannot), financial preparation (no or unsure), financial law knowledge (do not know). The continuous variables were time (months), age, education (1–5), self-rated health, sedentary activity hours per day, diet behaviors and literacy (14–42), cognitive function (MoCA score 0–30), depressive symptoms (CES-D score 0–30), adaption strategies (selection, optimization, compensation), coping strategies (action and positive thinking, negative, emotion-focused), family social support (4–12), friend social support (4–12), and life satisfaction (1–5). Note 2: ^#^ Only those who had family were included in this model. Note 3: The analysis was conducted by generalized estimating equation with normal or binomial distribution models. # QIC is abbreviated for the statistic value: quasi information criterion. * *p* < 0.05, ** *p* < 0.01, *** *p* < 0.001.

## References

[1] Rowe J.W., Kahn R.L., Rowe J.W., Kahn R.L. (1997). The structure of successful aging. Successful Aging.

[2] Adams G.A., Rau B.L. (2011). Putting off tomorrow to do what you want today: Planning for retirement. Am. Psychol..

[3] Depp C.A., Jeste D.V. (2006). Definitions and predictors of successful aging: A comprehensive review of larger quantitative studies. Am. J. Geriatr. Psychiatr..

[4] Donnelly E.A., Hinterlong J.E. (2010). Changes in social participation and volunteer activity among recently widowed older adults. Gerontologist.

[5] Elwood P., Galante J., Pickering J., Palmer S., Bayer A., Ben-Shlomo Y., Longley M., Gallacher J. (2013). Healthy lifestyles reduce the incidence of chronic diseases and dementia: Evidence from the Caerphilly cohort study. PLoS ONE.

[6] Hassan M.K., Lawrence S.B. (2007). Retirement savings of the hip generation: A study of retirement preparation among individuals in their fifties. Southwest. Econ. Rev..

[7] Hsu H.C., Chang W.C. (2015). Reducing the risks of morbidity, disability, and mortality using successful aging strategies. J. Am. Geriatr. Soc..

[8] Lennartsson C., Silverstein M. (2001). Does engagement with life enhance survival of elderly people in Sweden? The role of social and leisure activities. J. Gerontol. B Psychol. Sci. Soc. Sci..

[9] McLaughlin D., Vagenas D., Pachana N.A., Begum N., Dobson A. (2010). Gender differences in social network size and satisfaction in adults in their 70s. J. Health Psychol..

[10] Pollard C., Kennedy P. (2007). A longitudinal analysis of emotional impact, coping strategies and post-traumatic psychological growth following spinal cord injury: A 10-year review. Br. J. Health. Psychol..

[11] Pruchno R.A., Wilson-Genderson M., Rose M., Carwright F. (2010). Successful aging: Early influences and contemporary characteristics. Gerontologist.

[12] Estebasari F., Taghdisi M.H., Foroushani A.R., Ardebili H.E., Shojaeizadeh D. (2014). An educational proram based on the successful aging approach on helaht-promoting behaviors in the elderly: A clinical trial study. Iran. Red. Crescent Med. J..

[13] Hsu H.C., Chuang S.H., Hsu S.W., Tung H.J., Chang S.C., Lee M.M., Wang J.Y., Kuo L.T., Tseng F.Y., Po A.T. (2017). Evaluation of a successful aging promotion intervention program for middle-aged adults in Taiwan. Glob. Health Promot..

[14] Ngandu T., Lehtisalo J., Solomon A., Levälahti E., Ahtiluoto S., Antikainen R., Bäckman L., Hänninen T., Jula A., Laatikainen T. (2015). A 2 year multimodmain intervention of diet, exercise, cognitive training, and vascular risk monitoring versus control to prevent cognitive decline in at-risk elderly people (FINGER): A randomized controlled trial. Lancet.

[15] Rowe J.W., Kahn R.L. (2015). Successful aging 2.0: Conceptual expansions for the 21st century. J. Gerontol. B Psychol. Sci. Soc. Sci..

[16] Wilson M.L., Strayer T.E., Davis R., Harden S.M. (2018). Use of an integrated research-practice partnership to improve outcomes of a community-based strength-training program for older adults: Reach and effect of Lifelong Improvements through Fitness Together (LIFT). Int. J. Environ. Res. Public Health.

[17] Koniak-Griffin D., Brecht M.-L., Takayanagi S., Villegas J., Melendrez M., Balcázar H. (2015). A community health worker-led lifestyle behavior intervention for Latian (Hispanic) women: Feasibility and outcomes of a randomized controlled trial. Int. J. Nurs. Stud..

[18] Ockene I.S., Tellez T.L., Rosal M.C., Reed G.W., Mordes J., Merriam P.A., Olendzki B.C., Handelman G., Nicolosi R., Ma Y. (2011). Outcome of a Latino community-based intervention for the prevention of diabetes: The Lawrence Latino Diabetes Prevention Project. Am. J. Public Health.

[19] Connelly J., Kirk A., Masthoff J., MacRury S. (2013). The use of technology to promote physical activity in type 2 diabetes management: A systematic review. Diabet. Med..

[20] Bock C., Jarczok M.N., Litaker D. (2014). Community-based efforts to promote physical activity: A systematic review of interventions considering mode of delivery, study quality and population subgroups. J. Sci. Med. Sport.

[21] Foy C.G., Vitolins M.Z., Case L.D., Harris S.J., Massa-Fanlae C., Hopley R.J., Gardner L., Rudiger N., Yamamoto K., Swain B. (2013). Incorporating prosocial behavioral to promote physical activity in older adults: Rationale and design of the Program for Active Aging and Community Engagement (PACE). Contemp. Clin. Trials.

[22] Hefler M., Freeman B., Chapman S. (2013). Tobacco control advocacy in the age of social media: Using Facebook, Twitter and Change. Tob. Control.

[23] Burton E., Lewin G., Clemson L., Boldy D. (2013). Effectiveness of a lifestyle exercise program for older people receiving a restorative home care services: A pragmatic randomized controlled trial. Clin. Interv. Aging..

[24] Liu Y.M., Tsui C.M. (2014). A randomized trial comparing Tai Chi with and without cognitive-behavioral intervention (CBI) to reduce fear of falling in community-dwelling elderly people. Arch. Geriatr..

[25] Foroushani A.R., Estebsari F., Mostafaei D., Ardebili H.E., Shojaeizadeh D., Dastoorpour M., Jamshidi E., Taghdisi M.H. (2014). The effect of health promoting intervention on healthy lifestyle and social support in elders: A clinical trial study. Iran. Red. Crescent Med. J..

[26] Ferry M., Coley N., Andrieu S., Bonhomme C., Caubere J.P., Cesari M., Gautry J., Garcia Sanchez I., Hugonot L., Mansuy L. (2014). How to design nutritional intervention trials to slow cognitive decline in apparently healthy population and apply for efficacy claims: A statement from the International Academy on Nutrition and Aging Task Force. J. Nutr. Health Aging.

[27] Suzuki H., Kuraoka M., Yasunaga M., Nonaka K., Sakurai R., Takeuchi R., Murayama Y., Ohba H., Fujiwara Y. (2014). Cognitive intervention through a training program for picture book reading in community-dwelling older adults: A randomized controlled trail. BMC Geriatr..

[28] Hötting K., Holzschneider K., Stenzel A., Wolbers T., Röder B. (2013). Effects of a cognitive training on spatial learning and associated functional brain activations. BMC Neurosci..

[29] Wilcox S., Parrot A.P., Baruth M., Laken M., Condrasky M., Saunders R., Dowda M., Evans R., Addy C., Warren T.Y. (2013). The faith, activity, and nutrition program. Am. J. Prev. Med..

[30] Ball K.K., Ross L.A., Roth D.L., Edwards J.D. (2013). Speed of processing training in the ACTIVE Study: How much is needed and who benefits?. J. Aging Health.

[31] Ball K., Berch D.B., Helmers K.F., Jobe J.B., Leveck M.D., Marsiskie M., Morris J.N., The ACTIVE Study Group (2002). Effects of cognitive training interventions with older adults: A randomized controlled trial. J. Am. Med. Assoc..

[32] Kueider A.M., Parisi M., Gross A.L., Rebok G.W. (2012). Computerized cognitive training with older adults: A systematic review. PLoS ONE.

[33] Angevaren M., Aufdemkampe G., Verhaar H.J., Aleman A., Vanhees L. (2008). Physical activity and enhanced fitness to improve cognitive function in older people without known cognitive impairment. Cochrane Database Syst. Rev..

[34] Sink K.M., Espeland M.A., Castro C.M., Church T., Cohen R., Dodson J.A., Guralnik J., Hendrie H.C., Jennings J., Katula J. (2015). LIFE Study Investigators. Effect of a 24-month physical activity intervention vs health education on cognitive outcomes in sedentary older adults: The LIFE randomized trial. JAMA.

[35] Fledderus M., Bohlmeijer E.T., Smit F., Westerhof G.J. (2011). Mental health promotion as a new goal in public mental care: A randomized controlled trial of an intervention enhancing psychological flexibility. Am. J. Public Health..

[36] Li K.Y., Hsu W.C., Lin L.J. (2014). Effect of the recreational life review program on patients with dementia in an outpatient clinic: A preliminary study. Percept. Mot. Skills.

[37] Gitlin L.N., Szanton S.L., Huang J., Roth D.L. (2014). Factors mediating the effects of a depression intervention on functional disability in older African Americans. J. Am. Geriatr. Soc..

[38] Crisp D., Griffiths K., Mackinnon A., Bennett K., Christensen H. (2014). An online intervention for reducing depressive symptoms: Secondary benefits for self-esteem, empowerment and quality of life. Psychiatr. Res..

[39] Sundsli K., Söderhamn U., Espnes G.A., Söderhamn O. (2014). Self-care telephone talks as a health-promotion intervention in urban home-living persons 75+ years of age: A randomized controlled study. Clin. Interv. Aging.

[40] Ramirezl E., Ortega A.R., Chamorro A., Colmenero J.M. (2014). A program of positive intervention in the elderly: Memories, gratitude and forgiveness. Aging Ment. Health.

[41] Adams K.B., Leibbrandt S., Moon H. (2011). A critical review of the literature on social and leisure activity and wellbeing in later life. Ageing Soc..

[42] Huxhold O., Fiori K.L., Windsor T.D. (2013). The dynamic interplay of social network characteristics, subjective well-being, and health: The costs and benefits of socio-emotional selectivity. Psychol. Aging.

[43] Chou Y.L., Hsu H.C., Chang-Lee S.N., Yang H.Y., Sung Y.H. (2014). Successful aging awareness and retirement preparation in a sample of medical center employees. Chung Shan Med. J..

[44] Lin J.P., Cheng S.T., Chi I., Fung H.H., Li L.W., Woo J. (2015). Life Satisfaction among older adults in Taiwan: The effects of marital relations. Successful Aging: Asian Perspectives.

[45] Proulx C.M., Snyder L.A. (2009). Families and Health: An Empirical Resource Guide for Researchers and Practitioners. Fam. Relat..

[46] Hopkinson J.B., Richardson A. (2015). A mixed-methods qualitative research study to develop a complex intervention for weight loss and anorexia in advanced cancer: The family approach to weight and eating. Palliat. Med..

[47] Boltz M., Resnick B., Chippendale T., Galvin J. (2014). Testing a family-centered intervention to promote functional and cognitive recovery in hospitalized older adults. J. Am. Geriatr. Soc..

[48] Reid R.D., McDonnell L.A., Riley D.L., Mark A.E., Mosca L., Beaton L., Papadakis S., Blanchard C.M., Mochari-Greenberger H., O’Farrell P. (2014). Effect of an intervention to improve the cardiovascular health of family members of patients with coronary artery disease: A randomized trial. CMAJ.

[49] Kim J., Kwon J., Anderson E.A. (2005). Factors related to retirement confidence: Retirement preparation and workplace financial education. J. Financ. Counsel. Plan. Educ..

[50] Hsu H.C. (2007). Exploring elderly people’s perspective on successful aging in Taiwan. Ageing Soc..

[51] UNECE Active Aging Group (2013). Project ‘Active Ageing Index (AAI)’ 2012. Concept, Methodology and Final Results.

[52] Alexander G.L., McClure J.B., Clavi J.H., Divine G.W., Stopponi M.A., Rolnick S.J., Heimendinger J., Tolsma D.D., Resnicow K., Campbell M.K. (2010). MENU Choices Team. A randomized clinical trial evaluating online interventions to improve fruit and vegetable consumption. Am. J. Public Health.

[53] Boekhout J.M., Berendsen B.A.J., Peels D.A., Bolman C.A.W., Lechner L. (2018). Evaluation of a computer-tailored healthy ageing intervention to promote physical activity among single older adults with a chronic disease. International. J. Environ. Res. Public Health.

[54] Anderson-Bill E.S., Winett R.A., Wojcik J.R. (2011). Social cognitive determinants of nutrition and physical activity among web-health users enrolling in an online intervention: The influence of social support, self-efficacy, outcome expectations, and self-regulation. J. Med. Internet Res..

[55] Balastouskas P., Kennedy C.M., Buchan I., Powell J., Ainsworth J. (2015). The role of social network technologies in online health promotion: A narrative review of theoretical and empirical factors influencing intervention effectiveness. J. Med. Internet Res..

[56] Bewick B.M., Rumball K., Birtwistle J.C., Shaw J.R., Johnson O., Raistrick D., Tober G. (2014). Developing a web-based intervention to increase motivation to change and encourage uptake of specialist face-to-face treatment by hospital inpatients: Change Drinking. Drug Alcohol Rev..

[57] Stratton E., Lampit A., Choi I., Calvo R.A., Harvey S.B., Glozier N. (2017). Effectiveness of eHealth interventions for reducing mental health conditions in employees: A systematic review and meta-analysis. PLoS ONE.

[58] Eaton L.H., Doorenbos A.Z., Schmitz K.L., Carpenter K.M., McGergor B.A. (2011). Establishing treatment fidelity in a web-based behavioral intervention study. Nurs. Res..

[59] Maher C., Ferguson M., Vandelanotte C., Plotnikoff R., de Boudeaudhuij I., Thomas S., Nelson-Field K., Olds T. (2015). A web-based, social networking physical activity intervention for insufficiently active adults delivered via Facebook App: Randomized controlled trial. J. Med. Internet Res..

[60] Morrell R.W., Mayhorn C.B., Echt K.V., Burdick D.C., Kwon S. (2004). Why older adults use or dot not use the Internet. Gerotechnology: Research and Practice in Technology and Aging: A Textbook and Reference for Multiple Disciplines.

[61] Pak R., McLaughlin A. (2011). Designing Displays for Older Adults.

[62] Folstein M.F., Folstein S.E., McHugh P.R. (1975). Mini-Mental State: A practical method for grading the state of patients for the clinician. J. Psychiatr. Res..

[63] Nagi S.Z. (1976). An epidemiology of disability among adults in the United States. Milbank Mem. Fund Q. Health Soc..

[64] Radloff L.S. (1977). The CES-D scale: A self-reported depression scale for research in the general population. Appl. Psychol. Meas..

[65] Nasreddine Z.S., Phillips N.A., Bédirian V., Charbonneau S., Whitehead V., Collin I., Cummings J.L., Chertkow H. (2005). The Montreal Cognitive Assessment (MoCA): A Brief Screening Tool for Mild Cognitive Impairment. J. Am. Geriatr. Soc..

[66] Baltes P.B., Baltes M.M., Baltes P.B., Baltes M.M. (1990). Psychological perspectives on successful aging: The model of selective optimization with compensation. Successful Aging: Perspectives from the Behavioral Sciences.

[67] Donnellan C., Hevey D., Hickey A., O’Neill D. (2012). Adaptation to stroke using a model of successful aging. Neuropsychol. Dev. Cogn. B Aging. Neuropsychol. Cogn..

[68] Carver C.S. (1997). You want to measure coping but your protocol’s too long: Consider the Brief COPE. Int. J. Behav. Med..

[69] Liang K.Y., Zeger S.L. (1986). Longitudinal data analysis using generalized linear models. Biometrika.

[70] Linnan L., Steckler A., Steckler A., Linan L. (2002). Process evaluation for public health interventions and research: An overview. Process Evaluation for Public Health Interventions and Research.

